# Oleylamine-Mediated Hydrothermal Growth of Millimeter-Long Cu Nanowires and Their Electrocatalytic Activity for Reduction of Nitrate

**DOI:** 10.3390/nano8040192

**Published:** 2018-03-27

**Authors:** Yifan Zheng, Nana Chen, Chunxiao Wang, Xiaoping Zhang, Zongjian Liu

**Affiliations:** Institute of Industrial Catalysis, College of Chemical Engineering, Zhejiang University of Technology, Hangzhou 300014, China; zhengyifan@zjut.edu.cn (Y.Z.); 2111601009@zjut.edu.cn (N.C.); 2111301176@zjut.edu.cn (C.W.); 2111501036@zjut.edu.cn (X.Z.)

**Keywords:** Cu nanowires, hydrothermal method, oleylamine, nitrate, electroreduction

## Abstract

While high-aspect-ratio metal nanowires are essential for producing nanowire-based electrodes of good performance used in electronics and electrocatalysis, the synthesis of millimeter-long Cu nanowires remains a challenge. This work demonstrates an oleylamine-mediated hydrothermal method for synthesis of Cu nanowires with an average diameter of ~80 nm and a length up to several millimeters. An investigation on the role of oleylamine in nanowire formation by mass spectroscopy, small angle X-ray diffraction and transmission electron microscopy reveals that oleylamine serves as a mild reducing agent for slow reduction of Cu(II) to Cu, a complexing agent to form Cu(II)-oleylamine complex for guiding the nanowire growth, as well as a surfactant to generate lamellar phase structure for the formation of nanowire bundles. The growth mechanism of these millimeter-long Cu nanowire bundles is proposed based on the experimental observations. Electrochemical measurements by linear sweep voltammetry indicate that the self-supported nanowire electrode prepared from as-formed Cu nanowire bundles shows high catalytic activity for electroreduction of nitrate in water.

## 1. Introduction

Because of their highly anisotropic shape and finite size effects, metal nanowires often possess many physical and chemical properties that significantly differ from those observed in their bulk counterparts. These novel properties allow them to be promising candidates for future applications in a variety of fields, for example, electronics and catalysis. For example, Cu nanowires (CuNWs) have recently emerged as one of the most fascinating electrode materials [1,2,3,4,5,6,7,8,9,10,11,12,13]. Their excellent electrical conductivity, lower price in comparison with Ag, and good catalytic activity make them ideal building blocks for conducting electrodes used in electronics [1,2,3,4,5,6,7,8,9,10] and electrocatalysis [11,12,13]. It has been reported that the performance of nanowire-based conducting films depends strongly on the aspect ratio of the nanowires used, and long nanowires with small diameter are essential for producing film of high transparency and good conductivity [14]. In the case of electrocatalysis, the morphology of the electrocatalysts plays an important role in determining their activities [15,16,17] and the electrodes prepared from long nanowires with small diameter often own a high specific surface area and thus a good electrocatalytic activity. For these reasons, the growth of ultra-long CuNWs has been a subject of great interest in recent years.

Up to now, the methods for synthesis of CuNWs can be divided into three classes. The first class involves the use of hard template, and CuNWs are obtained by filling the channel of the template with Cu and then removing the template [18]. The second class employs the organic additives, which serve as soft templates, capping agents or/and complexing agents in the growth of CuNWs [1,2,3,4,5,6,7,8,9,10,19,20,21,22,23,24,25,26]. The third class does not use any hard template or organic additives [27,28,29]. In this case, however, high vacuum or electric field is often required. To pursue long CuNWs, the second class, which can be subdivided into hydrothermal reduction, aqueous solution reduction and non-aqueous synthesis (see Table 1), has been found to be the simplest and most effective. Obviously, CuNWs with length up to several hundreds of microns can be readily produced by hydrothermal reduction or aqueous solution reduction. It has been reported that CuNWs with length of several millimeters can be obtained by hydrothermal reduction in the presence of octadecylamine at 120–180 °C [30]. Under similar reaction conditions, however, Mayousse et al. found that the length of Cu nanowires was hundreds of microns [6]. Therefore, the synthesis of millimeter-long CuNWs remains a challenge.

In this work, we present an oleylamine-mediated hydrothermal growth of CuNWs with a diameter of ~80 nm and a length up to several millimeters. Oleylamine, an organic compound widely used in the synthesis of nanomaterials [31,32,33,34], contains a long linear carbon chain with a C=C in the middle and an amino group at one end of molecule. This special molecular structure allows oleylamine to be a complexing agent, a surfactant, a capping agent, a reducing agent and/or a solvent in nanomaterials synthesis. In the case of preparing CuNWs, oleylamine has been employed in aqueous solution reduction [8], non-aqueous synthesis [9,10,26] and even hydrothermal reduction [7]. The key difference between the method reported by Pan and co-workers [7] and our work lies in the role of oleylamine in the growth of CuNWs. Rather than acting only as capping agent in Pan’s work, in our method oleylamine serves as a mild reducing agent for slow reduction of Cu(II) to Cu, a complexing agent for guiding the nanowire growth, as well as a surfactant to generate a lamellar phase structure for the formation of CuNW bundles. In addition, no other reducing agents were used in our work, making the growth slow and feasible for very long nanowire formation. The growth mechanism of these CuNW bundles was investigated by mass spectroscopy, small angle X-ray diffraction (SAXRD) and transmission electron microscopy (TEM). The as-formed CuNW bundles were used for fabrication of self-supported nanowire electrode (SNWE) via thermal annealing, and the electrocatalytic activity for nitrate reduction of the resulting SNWE was measured by linear sweep voltammetry (LSV).

## 2. Materials and Methods

All chemicals were purchased from Aladdin (Shanghai, China) and used without further purification. In a typical protocol for synthesis of CuNWs, 1.070 g of oleylamine and 0.135 g of anhydrous copper chloride was added to 80 mL of deionized water and then stirred vigorously for 4 h to obtain a blue emulsion. The emulsion was transferred into a Teflon-lined 100 mL autoclave and kept at 160 °C for 96 h. After the autoclave was cooled down to room temperature, the solid product was centrifuged and then washed with n-hexane, deionized water and ethanol, respectively. For comparison, CuNWs were also prepared by an ethylenediamine-mediated aqueous solution reduction method and the experimental details could be found in our previous work [23]. 

To prepare the SNWE, a CuNW film was first obtained by filling a mould with dense CuNW slurry and then removing the mould after the CuNW-containing mould was dried in N_2_ atmosphere at 70 °C for 10 h. The resulting CuNW film was connected with a Cu wire and then annealed under Ar atmosphere at a given temperature for 30 min. Finally, the Cu wire was sealed with epoxy resin, and a SNWE with a size of about 0.5 × 0.6 × 0.05 cm^3^ was obtained.

The morphology of the samples was examined using a scanning electron microscope (SEM, Hitachi S-4700, Hitachi High-Technologies Corporation, Tokyo, Japan) operating at 15 kV. The phase composition was analyzed by X-ray diffraction (XRD), which was performed on a Thermo ARL XTRA X-ray diffractometer (Thermo Fisher Scientific, Waltham, MA, USA) using Cu Kα X-ray source. The microstructure investigations were performed with a Tecnai G2 F30 S-Twin transmission electron microscopy (Thermo Fisher Scientific, Waltham, MA, USA) operating at 300 kV. The mass spectra were recorded on a Therm LCQ TM Deca XP plus trap mass spectrometer (Thermo Fisher Scientific, Waltham, MA, USA) with electrospray ion source operating in a positive mode. 

Electrochemical measurements were performed at room temperature with a CHI660E workstation (CH Instruments Inc., Shanghai, China). The setup was a conventional three-electrode cell with a platinum wire as counter electrode and a saturated calomel electrode (SCE) as reference electrode. LSV, conducted in acidic medium with 0.1 M Na_2_SO_4_ as supporting electrolyte, was used to characterize the electrocatalytic activity for nitrate reduction. Before measurement, N_2_ purging was performed to remove the oxygen from the electrolyte solution. 

## 3. Results and Discussion

### 3.1. Oleylamine-Mediated Hydrothermal Growth of Cu Nanowires

**Figure 1:**
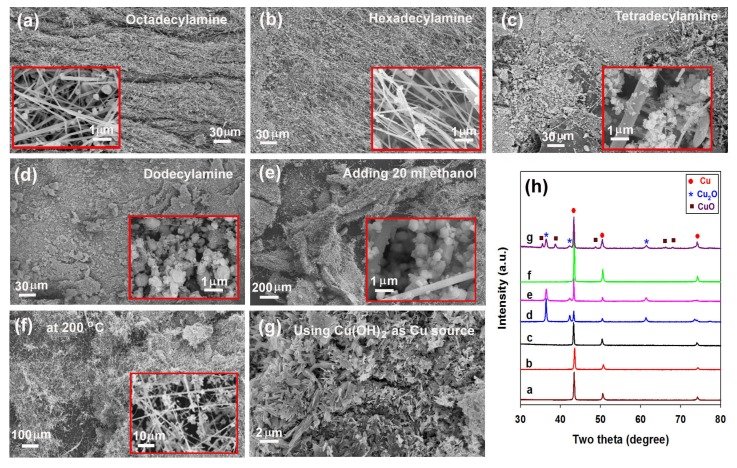
(**a**–**d**) Scanning electron microscope (SEM) images of the products formed at 160 °C for 96 h by replacing oleylamine with octadecylamine, hexadecylamine, tetradecylamine, or dodecylamine; (**e**–**g**) SEM images of the products formed in the presence of oleylamine for 96 h by adding 20 ml ethanol as reducing agent, changing reaction temperature from 160 °C to 200 °C, or replacing CuCl_2_ with Cu(OH)_2_ as Cu source; (**h**) X-ray diffraction (XRD) patterns of the products presented in Figure 1a–g. Insets of Figure 1a–f are high-magnification SEM images of the corresponding products.

**Figure 2:**
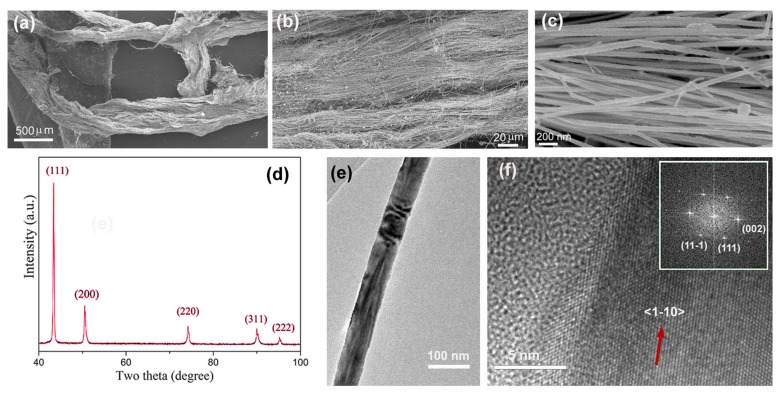
(**a**–**c**) SEM images at different magnifications of the product formed in the presence of oleylamine at 160 °C for 96 h; (**d**) XRD patterns of the product; (**e**,**f**) transmission electron microscopy (TEM) images of a nanowire. Inset of Figure 2f is the corresponding reduced fast Fourier transform image.

We tried to prepare long CuNWs under various conditions, for example, using different linear amines or Cu sources, operating at different temperatures and adding ethanol as reducing agent. Although very long CuNW bundles can be formed at 160 °C for 96 h with CuCl_2_ as Cu source and oleylamine as reducing agent, we cannot observe similar CuNW bundles by replacing oleylamine with other long-chain linear amines (see Figure 1a–d), adding ethanol as the reducing agent (Figure 1e), operating at 200 °C (Figure 1f), or using Cu(OH)_2_ as Cu source (Figure 1g), and in some cases CuO or/and Cu_2_O appear in the product (Figure 1h). Figure 2 presents the SEM images of the product formed at 160 °C for 96 h in presence of oleylamine. Low magnification SEM images of this sample indicate that the product is composed of bundles of nanowires with a length up to several millimeters (Figure 2a,b). High magnification SEM image (Figure 2c) reveals that these nanowires posses an average diameter of about 80 nm and thus the aspect ratio is estimated to be about 3 × 10^5^. The XRD pattern of the product is presented in Figure 2d. The peaks at 43.3°, 50.4°,74.1°, 89.9° and 95.1° can be indexed to Cu with a face-centered cubic (fcc) structure (JCPDS 04-0836) and correspond to the diffractions of (111), (200), (220), (311) and (222) planes, respectively. The TEM investigation confirms that these nanostructures are nanowires, rather than nanotubes (Figure 2e). The high resolution TEM image of a nanowire (Figure 2f), along with its reduced fast Fourier transform (FFT) image (inset of Figure 2f), suggests that the nanowire grows along the <111> direction.

**Figure 3:**
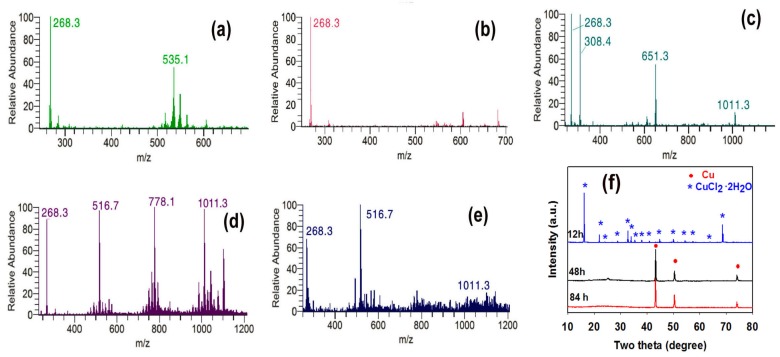
Mass spectra of oleylamine (**a**) and reaction mixtures after different reaction times: (**b**) 0 h (namely blue emulsion), (**c**) 12 h, (**d**) 48 h, and (**e**) 84 h; (**f**) XRD patterns of the product formed at different reaction times.

Although Guo et al. reported that, in the absence of water, CuCl_2_ could not be reduced to Cu by oleylamine even at a temperature of 300 °C [9], our above results demonstrate that under hydrothermal condition CuCl_2_ can be slowly reduced to Cu by oleylamine, leading to the formation of very long nanowire bundles. To understand the growth mechanism of the nanowires, mass spectrometry was first used to probe the role of oleylamine in the nanowire formation (Figure 3a–e). The mass spectrum of oleylamine displays two peaks, which can be attributed to the dimeric ion (*m*/*z* = 535.1) and the pseudomolecular ion (*m*/*z* = 268.3) originating from oleylamine molecule by addition of proton (Figure 3a). As indicated by Figure 3b, mixing oleylamine with CuCl_2_ solution and then stirring the mixture for 4 h at room temperature does not cause the formation of Cu(II)-oleylamine complex. However, after the mixture is heated at 160 °C for 12 h, a complex, namely Cu(oleylamine)_2_(OH)Cl, forms as confirmed by the peak at *m*/*z* = 651.2 (Figure 3c). Because the m/z value is very small, the new peak at *m*/*z* = 308.4 observed in Figure 3c is obviously not the molecular ion peak of any complex associated with oleylamine and CuCl_2_. Based on the result that it disappears when [Cu(oleylamine)_2_OH]Cl is completely consumed in the reaction (see Figure 3d), we speculate that the peak at *m*/*z* = 308.4 might be related to the fragment ion of [Cu(oleylamine)_2_OH]Cl. Besides the peaks at *m*/*z* = 651.2 and 308.4, a peak at *m*/*z* = 1011.3 can also be observed in Figure 3c. As the reaction proceeds, the intensity of the peak at *m*/*z* = 1011.3 increases (see Figure 3d). In addition, two new peaks appear at *m*/*z* = 516.7 and 778.1 (see Figure 3d). After 84 h of reaction (Figure 3e), the peak at *m*/*z* = 516.7 remains but those at *m*/*z* = 778.1 and 1011.3 almost disappear, implying that the first peak is associated with a reduction product but the latter two with reaction intermediates. The inference that the peak at *m*/*z* = 516.7 is related to a reduction product is also supported by the XRD patterns of the products formed at different reaction durations, where Cu crystal phase is observed when the peak at *m*/*z* = 516.7 appears (see Figure 3f). As suggested by Xia and co-workers in the synthesis of Au nanoparticles [33], the peak at *m*/*z* = 516.7 can be assigned to dioleylamine molecule, namely (C_8_H_17_-CH=CH-C_8_H_16_)_2_NH. They also found that, because of the aurophilic interaction between two Au(I) atoms, the AuCl-oleylamine complex polymers, for example, Au_2_(oleylamine)Cl, Au_2_(oleylamine)_2_Cl_3_, Au_5_(oleylamine)_5_Cl_3_ and Au_6_(oleylamine)_5_Cl_3_, were formed during the growth of Au nanowires [34]. Although the aurophilic interaction between two Cu(I) atoms is much weaker than that between two Au(I) ions, we speculate that similar polymers can be produced after Cu(II) is reduced to Cu(I) and the peaks at *m*/*z* = 1011.3 and *m*/*z* = 778.1 might be assigned to the polymers Cu_3_(oleylamine)_3_OH and Cu_3_(oleylamine)_2_(OH)Cl, respectively.

**Figure 4:**
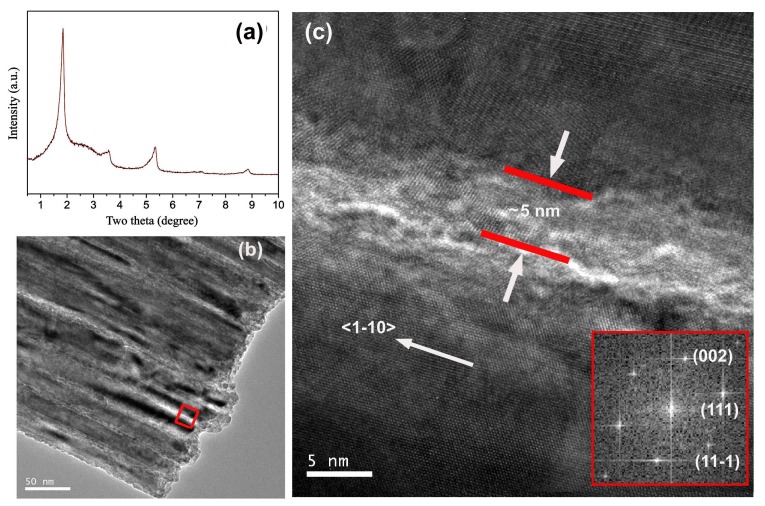
**Figure** **4.** (**a**) Small angle X-ray diffraction (SAXRD) for the product after 48 h of reaction; (**b**) TEM image of CuNW bundles observed in the product after 48 h of reaction; (**c**) An enlarged version of the framed region in Figure 4b. Inset of Figure 4c is the corresponding reduced FFT image.

The mass spectral analysis indicates that Cu^2+^ is reduced to Cu by oleylamine, probably via a pathway similar to that of Au^+^ to Au [33]. In the oleylamine-mediated growth of Au nanowires, a lamellar phase structure was found to be responsible for the formation of Au nanowire bundles [32,34]. To clarify whether a similar lamellar phase structure controls the growth of CuNW bundles, SAXRD was used to exam the product after 48 h of reaction. The diffraction pattern recorded exhibits five peaks with d-spacings of 4.794, 2.428, 1.655, 1.242 and 0.999 nm (see Figure 4a). These equally spaced peaks can be indexed as a periodic lamellar structure with layer spacing of ~4.8 nm, a value close to the width of an oleylamine bilayer. This finding hints that the growth of CuNW bundles is also governed by a lamellar phase structure. Although the growth of both CuNWs and Au nanowires are guided by a lamellar phase structure, the former possesses a much larger diameter than the latter (namely ~80 nm vs. ~2 nm). Even in the early growth stage, the diameter of CuNWs may reach about 20 nm (Figure 4b) despite that the separation between two neighboring CuNWs is about 5 nm (Figure 4c), a value close to ~4.8 nm found in small angle XRD. The possible reason for this difference is that the lamellar phase structure in the growth of Au nanowires originates from the aurophilic interaction between two Au(I) atoms [34] but that in our case should result from self-assembly of amphiphilic oleylamine molecules in the water, namely formation of lamellar micelles.

**Figure 5:**
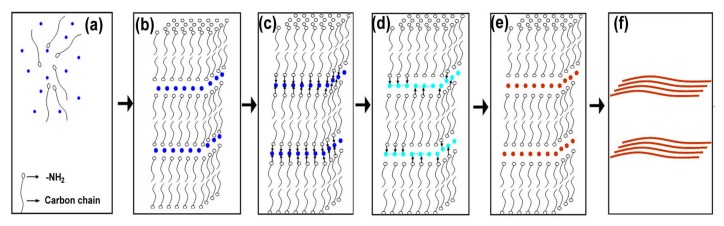
Schematic illustration of the growth of ultra-long Cu nanowire bundles: (**a**) oleylamine molecules disperse randomly in the water; (**b**) oleylamine molecules self-assembly into lamellar micelles; (**c**) Cu(oleylamine)_2_(OH)Cl complexes form within the micelles; (**d**) complex polymers, namely Cu_3_(oleylamine)_3_OH and Cu_3_(oleylamine)_2_(OH)Cl, form as Cu(II) is reduced to Cu(I); (**e**) Cu(I) species are further reduced to Cu; (**f**) nanowire bundles form. Arrows in Figure 7c,d represent the complexation. Cu(II), Cu(I), and Cu are indicated by blue, sky blue and red dots, respectively.

Based on the experimental results described above, a possible mechanism for the growth of ultra-long Cu nanowire bundles is proposed. Oleylamine molecules possess a hydrophilic head (namely –NH_2_) and a hydrophilic tail (namely carbon chain). At low concentrations, oleylamine molecules may disperse randomly in the water with their hydrophilic tails contacting with water (Figure 5a). To reduce the free energy of the system by decreasing the contact area of hydrophobic tails with water, the oleylamine molecules tend to self-assemble into lamellar micelles when the concentration of oleylamine is high (Figure 5b). The hydrophilic layer formed by the polar heads of oleylamine molecules allows Cu^2+^ ions (see deep blue dots in Figure 5a) to migrate from the aqueous phase into the lamellar micelles (Figure 5b). At high reaction temperatures (e.g., 160 °C), the hydrolysis of CuCl_2_ may occur and thus a Cu(II)-oleylamine complex, namely Cu(oleylamine)_2_(OH)Cl, forms within the lamellar phase structure (Figure 5c). Since the formation of Cu(II)-oleylamine complex, as well as reduction of CuCl_2_ to Cu by oleylamine, could not be observed in the absence of water [9], the partial hydrolysis of CuCl_2_ is believed to be crucial for the formation of Cu(II)-oleylamine complex and the reduction of Cu(II) to Cu. As the reaction proceeds, Cu (II) is reduced to Cu (I) (see sky blue dots in Figure 5d), and complex polymers, namely Cu_3_(oleylamine)_3_OH and Cu_3_(oleylamine)_2_(OH)Cl, form as a result of the aurophilic interaction between two Cu(I) atoms (Figure 5d). Cu(I) species within the complex polymers can be further reduced slowly to Cu (see red dots in Figure 5e). Because of the steric hindrance to some growing planes of Cu crystals exerted by oleylamine molecules, the reduction of Cu(I) species within the complex polymers favors the growth of CuNWs. In particular, the slow growth of CuNWs in such a lamellar template finally leads to the formation of ultra-long nanowire bundles (Figure 5f).

### 3.2. Self-Supported CuNW Electrodes and Their Electrocatalytic Activity for Nitrate Reduction

**Figure 6:**
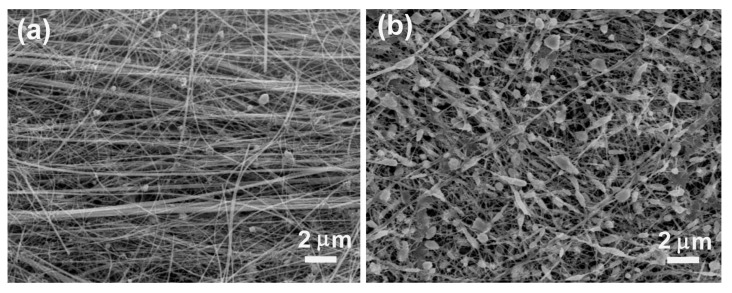
SEM images of the self-supported nanowire electrode (SNWE) prepared by thermal annealing of CuNW bundles at different temperatures: (**a**) 300 °C and (**b**) 400 °C.

Nitrate is one of the important contaminants found in surface and ground water. To deal with nitrate contamination, a number of methods, such as ion exchange, biological denitrification and electrochemical treatment, have been developed. Among these methods, the electroreduction of nitrate has drawn much attention due to its convenience and low cost [35,36,37,38,39]. In our previous work [13], we prepared SNWEs via thermal annealing of CuNWs synthesized by ethylenediamine-mediated aqueous reduction method (the average diameter of CuNWs is about 130 nm, and the electrode prepared is denoted as SNWE-1) and then studied the effect of annealing temperature on their electrocatalytic activity for reduction of nitrate. We found that, to produce a SNWE-1 with good physical stability, an annealing temperature of 600 °C or above was needed for the formation of a stable nanowire network via the melting of CuNWs [13], or else the as-prepared SNWE-1 deformed easily in aqueous solution (e.g., swelled or broke up), resulting in a sharp decay in its electrocatalytic activity. Therefore, further improvement of the electrocatalytic activity of these SNWEs cannot be achieved by lowering the annealing temperature. Herein, we try to use the as-formed nanowire bundles to fabricate SNWEs (denoted as SNWE-2) at low annealing temperatures and then make a comparison of electrocatalytic activity for nitrate reduction between SNWE-1 and SNWE-2. Interestingly, we find that the as-prepared SNWE-2 is physically stable and does not deform in the solution even though the annealing temperature is 300 °C. The SEM image of the SNWE-2 prepared at 300 °C demonstrates that the melting of CuNWs doesn’t occur at an annealing temperature of 300 °C (see Figure 6a). This implies that the physical stability of the SNWE-2 prepared at 300 °C cannot be attributed to the melting of CuNWs as observed at an annealing temperature of 400 °C (see Figure 6b). We speculate that such a physical stability should be associated with the bundle structure of CuNWs, where CuNWs are long and tangled with each other. 

**Figure 7:**
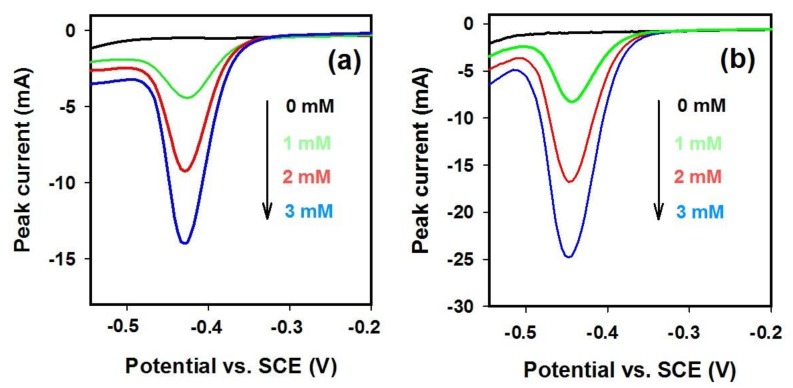
Linear sweep voltammetry (LSV) curves recorded in solutions containing 0.1 M Na_2_SO_4_ and nitrate of different concentrations (pH = 2) at two physically stable electrodes: (**a**) SNWE-1 prepared at 600 °C and (**b**) SNWE-2 prepared at 400 °C. The scan rate is 40 mV s^−1^.

The electroreduction of nitrate at the physically stable SNWE-2 prepared at 400 °C was studied by LSV. Since the SNWEs are stable under acidic conditions [13], the electroreduction of nitrate was conducted in an acidic medium (pH = 2), where nitrate ions are reduced to ammonium ions at copper surface according to the equation NO_3_^−^ + 10 H^+^ + 8 e = 3 H_2_O + NH_4_^+^ [40]. For comparison, a physically stable SNWE-1 with a size of about 0.5 × 0.6 × 0.05 cm^3^, prepared at an annealing temperature of 600 °C, was also investigated. LSV curves were recorded at 40 mVs^−1^ for solutions containing different concentrations of nitrate. Figure 7 presents the LSV curves observed at two electrodes. At both two SNWEs, we can observe a well-shaped reduction peak with its current increasing with the nitrate concentration. At the same nitrate concentration, the peak current observed at SNWE-2 is about 2 times higher than that at SNWE-1, suggesting that SNWE-2 owns a much higher electrocatalytic activity for nitrate reduction than SNWE-1. The much better performance observed at SNWE-2 can be interpreted in terms of the fact that the diameter of CuNWs used for preparation of SNWE-2 is smaller than that for SNWE-1. As a result, a higher specific surface area is expected for SNWE-2 and thus SNWE-2 is more electrochemically accessible for nitrate ions in comparison with SNWE-2. The above result also demonstrates that, by using the ultra-long CuNW bundles as building blocks, the performance of the SNWE can be greatly improved.

## 4. Conclusions

In summary, CuNWs with an average diameter of ~80 nm and a length up to several millimeters (the aspect ratio is about 3 × 10^5^) has been synthesized via an oleylamine-mediated hydrothermal method. In the growth of CuNWs, oleylamine plays multiple roles, serving as a surfactant to generate lamellar phase structure, a complexant to form Cu(II)/Cu(I)-oleylamine complex, as well as a mild reducing agent to reduce Cu(II) or Cu(I) slowly. The millimeter-long nanowire bundles are believed to be a comprehensive result from several factors: the lamellar phase structure as template for nanowire bundle growth, the Cu(II)/Cu(I)-oleylamine complex governing the anisotropic growth of Cu crystals, and a slow growth rate favoring the formation of very long nanowires. These millimeter-long nanowire bundles are ideal building blocks for preparation of physically stable SNWE used as cathode for electroreduction of nitrate in water.

## Figures and Tables

**Table 1:** Methods for synthesis of long Cu nanowires (CuNWs) in the presence of organic additives.

Method	Cu Source	Solvent	Reducing Agent	Other Organics Added	Reaction Temperature and Duration	Length of CuNWs	Refs.
Hydrothermal reduction	CuCl_2_	water	glucose	hexadecylamine	120°C for 2 h	several microns	[13]
	CuCl_2_	water	glucose	hexadecylamine	120°C for 12 h	tens of microns	[5]
	Cu(NO_3_)_2_	water	ethylene glycol	polyvinyl pyrrolidone	160°C for 24 h	tens of microns	[20]
	CuCl_2_	water	octadecylamine	-	165°C for 140 h	hundreds of microns	[6]
	CuCl_2_	water	glucose	ethanol, oleic acid, and oleylamine	116 °C for 2–12 h	tens of microns	[7]
Aqueous solution reduction	Cu(NO_3_)_2_	water	hydrazine	ethylenediamine	40–80 °C for 25 min–15 h	tens of microns	[1,2,4,21,22]
	Cu(OH)_2_	water	hydrazine	ethylenediamine	70 °C for 1 h	tens of microns	[23]
	Cu(NO_3_)_2_	water	hydrazine	propanediamine	80 °C for 1 h	tens of microns	[24]
	CuCl_2_	water	glucose	hexadecylamine	100 °C for 6 h	hundreds of microns	[25]
	CuCl_2_	water–alcohol	L-ascorbic acid	oleylamine	55–85 °C for 12 h	hundreds of microns	[8]
Non-aqueous synthesis	CuCl	oleylamine	via disproportionation reaction	-	200 °C 30 min	tens of microns	[26]
	CuCl_2_	oleylamine	in the presence of catalytic Ni^2+^	-	175 °C for 10 h	tens of microns	[9]
	Cu(acac)_2_	hexadecylamine	in the presence of catalytic Pt	cetyltriamoninum bromide	180 °C for 10 h	hundreds of microns	[3]
	CuCl_2_	oleylamine	tris(trimethylsilyl)silane	oleic acid	165 °C for 10 h	tens of microns	[10]
